# Intensively Cultivated Landscape and *Varroa *Mite Infestation Are Associated with Reduced Honey Bee Nutritional State

**DOI:** 10.1371/journal.pone.0153531

**Published:** 2016-04-12

**Authors:** Adam G Dolezal, Jimena Carrillo-Tripp, W. Allen Miller, Bryony C. Bonning, Amy L. Toth

**Affiliations:** 1 Department of Ecology, Evolution, and Organismal Biology, Iowa State University, Ames, IA, United States of America; 2 Department of Plant Pathology and Microbiology, Iowa State University, Ames, IA, United States of America; 3 Department of Entomology, Iowa State University, Ames, IA, United States of America; Federal University of Viçosa, BRAZIL

## Abstract

As key pollinators, honey bees are crucial to many natural and agricultural ecosystems. An important factor in the health of honey bees is the availability of diverse floral resources. However, in many parts of the world, high-intensity agriculture could result in a reduction in honey bee forage. Previous studies have investigated how the landscape surrounding honey bee hives affects some aspects of honey bee health, but to our knowledge there have been no investigations of the effects of intensively cultivated landscapes on indicators of individual bee health such as nutritional physiology and pathogen loads. Furthermore, agricultural landscapes in different regions vary greatly in forage and land management, indicating a need for additional information on the relationship between honey bee health and landscape cultivation. Here, we add to this growing body of information by investigating differences in nutritional physiology between honey bees kept in areas of comparatively low and high cultivation in an area generally high agricultural intensity in the Midwestern United States. We focused on bees collected directly before winter, because overwintering stress poses one of the most serious problems for honey bees in temperate climates. We found that honey bees kept in areas of lower cultivation exhibited higher lipid levels than those kept in areas of high cultivation, but this effect was observed only in colonies that were free of *Varroa* mites. Furthermore, we found that the presence of mites was associated with lower lipid levels and higher titers of deformed wing virus (DWV), as well as a non-significant trend towards higher overwinter losses. Overall, these results show that mite infestation interacts with landscape, obscuring the effects of landscape alone and suggesting that the benefits of improved foraging landscape could be lost without adequate control of mite infestations.

## Introduction

Honey bees are economically-important managed pollinators, contributing billions of dollars in added yields to a variety of crops worldwide [1]. However, recent years have seen increases in annual honey bee colony losses and an increase in the costs associated with maintaining beekeeping operations [2, 3]. Significant efforts devoted to identifying the causes of these honey bee losses have led to the consensus that multiple environmental factors interact to stress honey bee colonies. These factors include exposure to a variety of pesticides, incidence of new and more widespread pathogens, increasing pressure from *Varroa destructor* mite infestations, and changes in landscape use that affect the floral resources available to honey bee foragers [4].

Nutrition has been identified as a major issue in honey bee health [5], as honey bee nutritional stress can exacerbate other stressors, increasing sensitivity to pesticides [6] and susceptibility to pathogens [7, 8]. Honey bees rely on plant resources in their environment for both nectar, which provides carbohydrates and becomes honey, and pollen, which provides all other nutritional factors, including protein, lipids, and micronutrients [9]. However, not all plants produce the same quantity of nectar or pollen, or provide equal nutritional quality, and a diverse floral diet can be important for bee survival and pathogen resistance [8, 10, 11]. Recent years have also seen significant shifts in landscape use, as natural habitats are displaced by more intensive agricultural landscapes [12]. This has been especially notable in areas such as the Midwestern USA, where there is extremely intense cultivation of crops that are not typically seen as forage for bees (i.e., corn and soybeans) along with highly effective control of weedy plant species that bees utilize as forage [13, 14]. The replacement of native and weedy plant resources due to agricultural intensification has dramatically changed the bee foraging landscape [12, 15].

Pollen source and availability have been implicated as major influences on pollinator nutrition and an important component of their declines [4, 16]. As the primary source of most nutrients, pollen diet affects a variety of processes and life-history traits, such as lifespan [11], behavioral maturation [17], immune response [18–20], pathogen resistance [7, 8], and pesticide sensitivity [6], and results in physiological changes in individual bees such as reduced vitellogenin levels [21, 22], hypopharyngeal gland size and quality [7, 23], protein [8], and lipid content [17]. Lipid content is of primary importance in the function of the fat body, an organ that not only stores fat, but plays a critical role in protein synthesis and storage, iron metabolism, immune response, detoxification, behavioral regulation [22, 24, 25], and preparation of bees for overwintering [26, 27]. Therefore, dietary effects on fat body size and overall lipid content are of particular interest.

Pathogen exposure is another major player in honey bee losses [28, 29], which can be exacerbated by poor nutrition [8, 18]. Honey bees are also frequently infested with the ectoparasitic *Varroa destructor* mite, which causes a variety of problems and is arguably the greatest current threat to honey bee health in much of the world. *Varroa* mites feed primarily upon pupal honey bees, consuming hemolymph and reproducing under the pupal cap [30]. Adult honey bees that were parasitized during development show numerous physiological repercussions, including lower water weight, protein content, and carbohydrate content, as well as physical deformities [31]. *Varroa* mites also transmit several detrimental bee viruses [30, 32], which have been implicated as players in Colony Collapse Disorder (CCD) and other large-scale bee losses [4, 28]. On a colony scale, *Varroa* infestation reduces pupal weight, though nutritional physiology in these colonies has been found to be highly variable [33]. Hence, the picture of how *Varroa* infestation affects nutritional physiology is incomplete.

Recent studies have attempted to evaluate the impacts of different regional landscapes on a variety of facets of honey bee health. For example, in the United Kingdom, the protein content of stored pollen (beebread) in a hive is negatively correlated with agricultural land use [34], and honey bees in rural areas preferentially forage on non-agricultural plants in rural landscapes [35]. In Kenya, proximity of honey bee hives to forests was correlated with higher honey yields [36]. Honey bee hives in France intensively forage on mass-flowering oilseed rape and sunflower, though they are most successful when kept in more forested areas [37]. Conversely, a study in Ohio found that hive-level metrics of colony health, like food storage and wax production, were positively correlated with agricultural land use instead of forest, possibly due to the use of non-crop weeds in field edges [38]. Overall, these studies show that it is difficult to draw broad conclusions from individual regions due to differences in agricultural management and forage types, and evaluating honey bee health in a variety of situations is necessary to gain a broader understanding of how agricultural landscape use affects honey bee heath. Furthermore, there is little information on how landscape affects honey bee nutritional physiology. This represents an important gap in our knowledge of how land use, forage, and bee health are connected.

One of the most critical components of the honey bee life cycle in temperate climates is overwintering [26, 39]. In the late autumn, the last generations of adult workers that emerge become physiologically and behaviorally distinct from the workers produced during the spring and summer. Spring and summer workers are short-lived (~30 days), whereas bees emerging in the autumn, called “winter bees” can live up to 8 months [40]. Instead of nursing brood and then transitioning to foraging as summer bees do, winter bees remain primarily in the nest and form a thermoregulatory cluster during cold weather, and then make up the workforce in the spring [41]. Therefore, the health of these ‘winter bees’ is of the utmost importance for the survival of a honey bee colony, and high lipid stores are one indicator of overwintering potential. Due to a variety of stressors [42–45], overwintering losses have increased substantially in the last few decades [46]. Therefore, a robust and healthy population of winter bees at the end of the autumn is critical to a colony’s ability to withstand the stress of a long winter [19, 26].

Here, we sought to better understand how the landscape surrounding honey bee hives in agriculturally-intensive areas was related to the physiology and virus load of pre-overwintering bees in Iowa, in the Midwestern USA. This area is of particular interest for pollinator health [47], because Iowa is cultivated intensively (> 80% of the area of the state) with monoculture crops of little known value to pollinators (e.g. corn and soybeans). To address the question of how landscape affects bee health, we sampled bees in mid-autumn, when colonies should be populated with “winter” bees [48], from apiaries kept by a diverse group of volunteer beekeepers throughout Iowa, USA. For each apiary sampled, we calculated the percent cultivated land surrounding the apiary, measured *Varroa* mite infestation levels, virus levels, protein content, and lipid stores of sampled bees. The goals of the study were to test the following hypotheses: 1) due to reduction in floral forage availability, intensively cultivated landscapes are associated with poor bee pre-overwintering nutritional physiology, 2) because poor pollen nutrition is associated with decreased immune response [18] and pathogen replication [8], intensively cultivated landscapes are associated with higher virus loads, 3) the presence of parasitic *Varroa* mites is associated with poor bee nutrition, and 4) the presence of *Varroa* mites is associated with higher virus loads.

## Methods

### Identification of Focal Sites

Potential apiaries were identified through the member directory of the Iowa Honey Producers’ Association (IHPA), Iowa’s state-wide beekeeping organization. Addresses of members were then mapped and the landscape surrounding their addresses was determined via ArcMap 10 by the Iowa State University GIS Support and Research Facility based on 2006 landscape data from the National Land Cover Database (NLCD). Addresses were geocoded using StreetMap North America (Esri). To ensure the privacy of participants, geographical coordinates of these sites are not reported, though county location is provided here (S1 Table). Landscape buffers were then analyzed at 5 mile (8.06 km) and 1 mile (1.61 km) radii to encompass the maximum foraging range [49] surrounding each apiary. Landscape cover was calculated as ‘cultivated’ (land used for the cultivation of agricultural plants, predominantly corn or soybeans) or ‘uncultivated’ (land not used for agricultural cultivation: forest, shrub, grassland, woody wetland, emergent herbaceous wetland, pasture/hay, open water, developed land (urban), barren land; see S1 Table for breakdown of each site.

After identification of landscape compositions, beekeepers with at least 2 hives/apiary and less than 50 hives/apiary were invited to participate in the study. We confirmed that apiaries were located at the address indicated in the IHPA directory; if not, the site of the apiary was re-mapped and landscape of the apiary was determined. To focus only on extreme differences in landscape composition, we sought to use only those sites that were in areas of very high cultivation or very low cultivation. To do this, we chose only sites in the top or bottom quartiles of cultivation percentage from among respondent beekeepers. This resulted in a “low cultivation” group (n = 12 apiaries), where 9–50% of the surrounding 8.0 km (5 mi.) radius (landscape was cultivated, and a “high cultivation” group (n = 11 apiaries), where 44–91% of the surrounding landscape was cultivated. In all cases, the proportion of cultivated land was similar at the 5 mile and 1 miles buffers (i.e., the apiary would fall in the same quartile at that analysis range).

### Collection of Samples from Cooperator Beekeepers

In October 2013, beekeepers were given instructions on how to collect samples. Then, each beekeeper was mailed a queen battery shipping box (JZBZ Honey Co.) containing detailed written instructions on the collection protocol, a small measuring cup, a small sponge, and a pre-paid shipping label. As nutrition and nutritional needs [26] and virus titers [50] are variable throughout the year, all bees were sampled during a 2 week time period. At a point during the last week of October or the first week of November, 2013, beekeepers were asked to collect approximately 100 mL of bees from the center of the nest (filling the provided measuring cup), separately from each hive, and pour these bees into the provided shipping box with a moistened sponge, and mail the container the next day. All shipments arrived within 36 h. Upon arrival, shipping boxes were inspected to ensure that bees were alive and in good condition; in all cases, >50% of the bees had survived; the vast majority contained very few dead bees. Boxes were then placed in a -80°C freezer for at least 12 hours. When bees are placed in the freezer, the majority of the live bees clustered in the top of the container; to ensure only live bees were used for analysis, we only sampled from the bees in these clusters. These frozen bees were then transferred to 50 mL centrifuge tubes for long term storage at -80°C. This method of sacrificing the bees is very fast, and care was taken to ameliorate any suffering. At the end of the next beekeeping season, volunteer beekeepers were contacted again and information was collected on overwintering success and mite treatment regimens.

### Sample Preparation, Protein Quantification, Lipid Quantification, and Body Mass Measurements

To ensure a representative sample of bees from each hive, approximately 40 mL of frozen bees were pulverized in liquid nitrogen with a ceramic mortar and pestle. Any remaining bees were set aside for *Varroa* quantification, resulting in a final analyzable sample size of n = 26 “low cultivation” hives and n = 24 “high cultivation” hives. From the homogenate, two subsamples of 0.25 g were taken. One sample was processed for lipid quantification using a phospho-vanillin spectrophotometric assay commonly used on honey bees [17]. Lipid per gram of bee was calculated based on the mass of the sample. From the original sample bees, 5 were randomly selected, completely dehydrated in a drying oven for 24 h at 55°C, and weighed, providing an average dry mass. The other sample from the homogenate was used to measure total protein content. Protein was extracted from powered bee tissue as described in Mutti et al., [51]. The protein extraction protocol was repeated on each sample four times, with a final sample analysis made by using an equal volume of each extraction to make a representative sample. Extracted protein was then analyzed by Bradford assay using albumin as a standard [52].

### Virus Quantification

Total RNA was extracted from approximately 0.25 g of bee homogenate (approximately the mass of 2–3 adult honey bees) using TRIzol (Life Technologies), treated with DNAse I, and diluted to 100 ng/ul. Genome copy number of black queen cell virus (BQCV), Israeli acute paralysis virus (IAPV), deformed wing virus (DWV), and sacbrood virus (SBV) was determined using the protocol and primers described in Carrillo-Tripp et al [53]. In short, virus sequences of 90–200 nt were amplified in one step RTqPCR using the iTaq™ Universal SYBR® Green One-Step Kit (BioRad) following an absolute quantification approach [54] using 100 ng of total RNA per sample. Amplification with technical duplicates was performed in a CFX 384 thermocycler (BioRad). The final copy number (viral genome equivalents/100 ng RNA) of each virus in each sample was calculated by extrapolation to a standard curve made by serial dilution (1:10) of a viral fragment RNA used as reference. Data out of the dynamic range for each target were considered below detection limits and treated as a titer of zero. The minimum limit of detection for SBV and IAPV was 4.92E+02 viral genome equivalents and for DWV and SBV 4.92E+03 viral genome equivalents [53].

### Mite Quantification

To test levels of mite infestation, we only used samples that contained at least 20 bees after lipid sample homogenization, which resulted in the exclusion of some of the samples provided by beekeepers. Overall, this resulted in 50 hives fit for analysis. While 20 bees is lower than ideal for mite counts [55], this was necessary due to the low number of bees provided by some beekeepers and the need to preserve the majority of bees for other analyses. The available frozen bees from each sample were washed in alcohol, shaken through a sieve until no more mites detached (at least two washes). Mites and bees were then counted and infestation was calculated as mites/bee (infestation rate). Samples were then designated as “mite infested” (with 1 or more mites in the sample) or “mite free” (zero mites).

### Statistical Analysis

Comparisons of lipid content, protein content, body mass, and virus titer were performed using a mixed linear effects model using the ‘lme’ function from the R package ‘nlme’. For each variable, effects due to landscape group, *Varroa* presence, and an interaction between the two were analyzed. The mixed linear effects model was then followed by an ANOVA. In cases with significant interactions between landscape group and *Varroa* presence, effect of landscape group was analyzed independently in the *Varroa* presence groups and vice versa. In all analyses, source beekeeper was used as a random effect. Comparison of mite infestation with overwinter loss, an apiary-level measure, was performed by creating an average lipid, protein, mass, and virus titer value for each apiary from the subsamples provided from it. Analysis of overwintering loss vs. each of these averaged factors and average mite infestation rate (mites/bee) was performed by linear regression using the ‘lm’ function in R. All samples fit normality and equal variance assumptions except virus titers and mite infestation rate. Both were log transformed to fit the equal variance assumption.

## Results

### *Varroa* Mite Presence and Relationship to Landscape Cultivation

Overall, samples of 50 hives from 22 apiaries were analyzed for *Varroa* infestation. At the time of sampling, 26 of these hives had undetectable mite levels. In all instances, at least one hive sample from each beekeeper had detectable mite levels. The mean infestation level was 6 mites/100 bees, ranging from approximately 1 to 16 mites/100 bees. There was no significant effect of landscape composition group on mite infestation level (F_1,20_ = 0.02, p>0.05) or on presence/absence of mites (Χ^2^(1, *N* = 50) = 0.334, *p* = 0.56).

### Relationship between Landscape Cultivation and *Varroa* Mites on Lipid Content, Protein Content, and Body Mass

**Fig 1:**
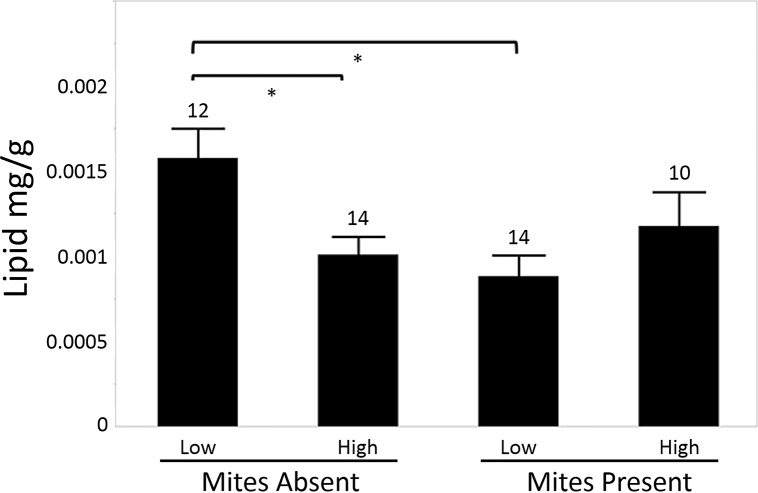
Relationship between landscape cultivation and lipid levels in absence of *Varroa* mite. Mean +/- SE of lipid concentration of bees from hives without mite infestations kept in low cultivation and high cultivation areas. Number of hives sampled indicated, * bracket indicate significant differences in lipid content

Colonies in which mites were detected showed significantly lower lipid levels (F_1,26_ = 4.77, p<0.05; Fig 1) than colonies without mites. When analyzing all samples, no differences were observed due to landscape group (F_1,20_ = 0.039, p>0.05), but a significant interaction was observed between landscape cultivation level and *Varroa* presence (F_1,26_ = 8.927, p<0.05). Due to this interaction, landscape group was analyzed independently in bees from colonies where *Varroa* were present or absent, and mite presence groups were contrasted independently within each landscape group. In bees from colonies with no mites detected, lipid content was higher in colonies from low vs. those in high cultivation landscapes (F_1,15_ = 8.43, p<0.05). In bees from colonies where mites were detected, however, no differences in lipid content were observed between bees from different landscape groups (F_1,12_ = 1.41, p>0.05). Furthermore, in bees from low cultivation areas, colonies with detectable *Varroa* levels had significantly lower lipid content (F_1,13_ = 12.07, p<0.005) while there were no differences due to mite infestation in colonies from the high cultivation landscape (F_1,13_ = 0.59, p>0.05).

There were no significant effects of landscape cultivation (F_1,20_ = 0.0085, p>0.05), *Varroa* detection group (F_1,26_ = 0.6170, p>0.05), or interaction of those variables (F_1,26_ = 0.0983, p>0.05) on protein content (S1 Fig). There were also no significant effects of landscape cultivation (F_1,20_ = 0.55, p>0.05), *Varroa* detection group (F_1,24_ = 0.48, p>0.05), or and interaction of those variables (F_1,24_ = 1.33, p>0.05) on dry body mass (S1 Fig).

### Relationship between Landscape Cultivation and *Varroa* Presence on Virus Titers

**Fig 2:**
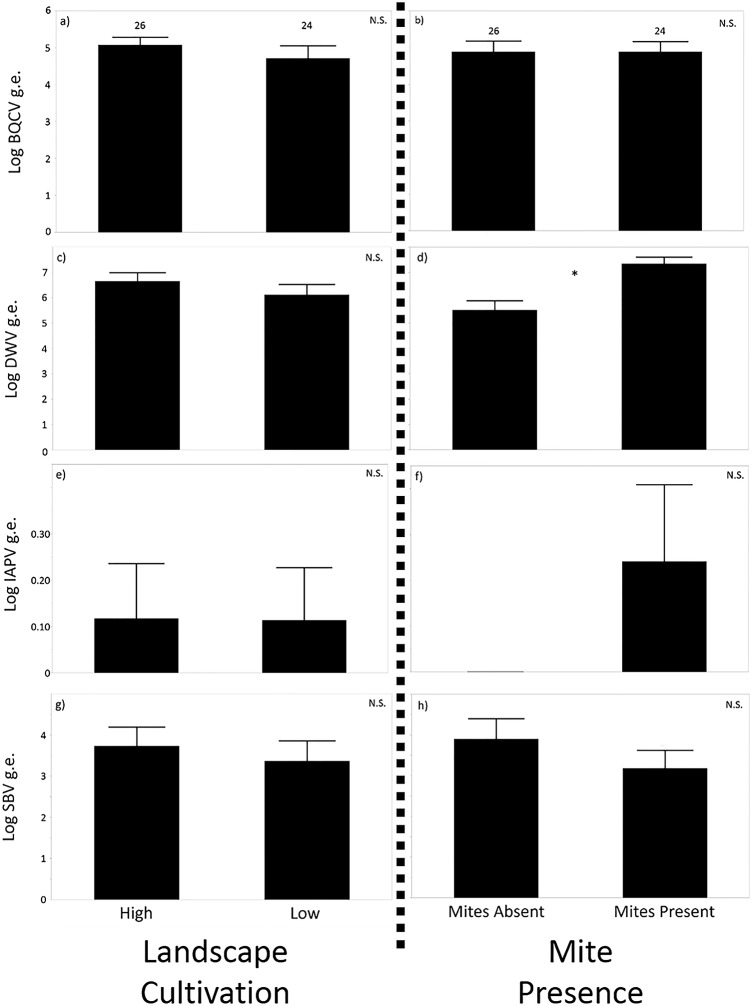
Virus levels in honey bees from low and high cultivation landscapes, in presence or absence of *Varroa* mite. Mean +/- SE of log transformed genome equivalents of BQCV (A, B), DWV (C, D), IAPV (E, F), and SBV (G, H) of bees from hives in low and high cultivation areas (A, C, E, G) and with *Varroa* mites absent or present (B, D, F, H). Number of hives sampled indicated, * indicates significant differences, N.S. denotes no significant differences.

There was no significant effect of landscape cultivation level, *Varroa* mite presence, or their interactions on BQCV, IAPV, or SBV levels (ANOVA, p>0.05; Fig 2A, 2C and 2D). There was no effect of landscape group (F_1,20_ = 1.29, p>0.05, Fig 2B) or an interaction between landscape group and *Varroa* presence (F_1,26_ = 0.21, p>0.05) on DWV levels. However, there was a significant main effect of *Varroa* presence (F_1,26_ = 14.47, p<0.0005) on DWV titers, with *Varroa*-infested bees exhibiting significantly higher titers (Fig 2C).

### Self-Reported Overwinter Losses

**Fig 3:**
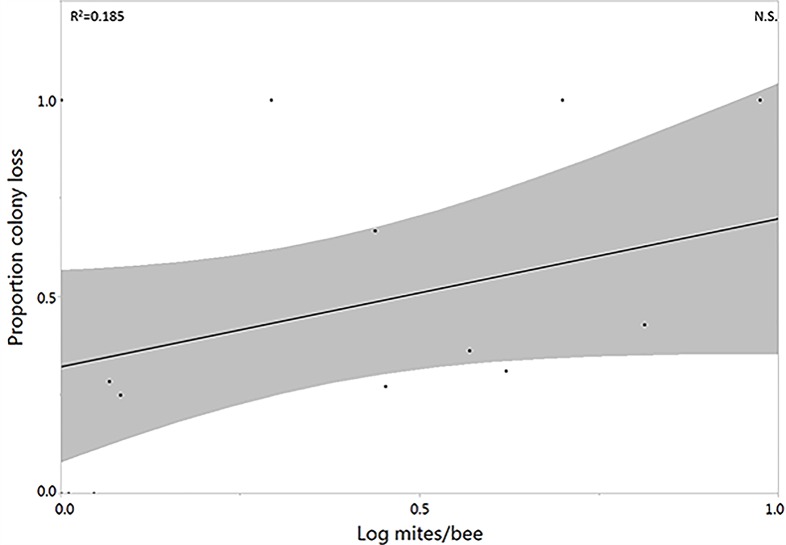
Relationship between apiary-average (from collected samples) *Varroa* mite infestation and apiary-average proportion hive losses. Shaded areas shows confidence of fit. N.S. denotes no significant correlation.

Interviews with beekeepers the following year were successfully conducted with 16 of 23 beekeepers, 8 with bees in low and 8 with bees in high cultivation landscapes. Of these, 5 had performed no chemical mite treatments in the year of our collections. Among those that did apply mite treatments, the treatment approach varied considerably, as did overwintering success. Of the responding beekeepers, estimated total overwintering losses after our sampling year averaged 49% (n = 16, S.E. = 9.53%). Individual hives were not tracked by the majority of beekeepers, so it was not possible to correlate individual metrics with overwinter losses. However, it was possible to contrast the apiary average values (generated from the samples provided) with overwinter losses for the limited number of respondent beekeepers. Landscape cultivation, *Varroa* presence, protein content, lipid content had no significant correlation with overwintering losses in these beekeepers (linear regression, p<0.05). The average mite infestation of each beekeepers’ sampled hives showed a positive, but not significant, trend with overwinter loss percentage (linear regression, R^2^ = 0.1271, F(1,14) = 3.184, p = 0.096; Fig 3).

## Discussion

The effects of heavily managed agricultural landscapes on honey bee health have been of increasing interest in recent years [16]. However, there has been little research on how different levels of cultivation affect honey bee nutritional physiology and pathogen load, nor how landscape and *Varroa* mite infestation interact to affect these factors. Here, we tested several hypotheses regarding the effects of foraging landscape and *Varroa* presence on nutritional physiology and virus infection. Our data support the hypothesis that bees kept in more intensively cultivated landscapes show poorer pre-overwintering nutritional physiology, as bees from our high cultivation landscape did show lower lipid levels than those from low cultivation areas. However, this was only the case in colonies without detectable *Varroa* infestation, as a significant interaction between *Varroa* presence and level of landscape cultivation was observed. Furthermore, our data support the hypotheses that *Varroa* infestation is associated with poorer nutritional physiology and with higher loads of at least one virus, DWV. Higher landscape cultivation was not, however, associated with any differences in virus titers, providing no support for the hypothesis that decreased pollen nutrition in high cultivation landscapes leads to elevated virus titers.

Honey bees from hives kept in areas of lower landscape cultivation possessed higher pre-overwintering lipid stores than those kept in more agriculturally intensive areas (Fig 1). This suggests that in the Iowa landscape, bees kept in areas of high cultivation experience lower forage quality that can significantly impact nutritional physiology. Furthermore, the bees sampled here represent some of the most important workers in a colony’s lifecycle–the winter bees. In late autumn, when collections were performed, honey bee colonies in temperate climates have ceased most brood rearing and food collection and are preparing for winter, producing many ‘winter bees’: long-lived individuals that maintain some of the physiological traits of younger bees [48]. Therefore, the lower lipid stores found in pre-overwinter bees from colonies in highly-cultivated areas could be a cause for concern. That said, we did not see a significant effect of landscape, *Varroa* infestation, or lipid content on apiary-average overwintering losses. However, this analysis may have inadequate power to detect these effects due to low sample size, potentially inaccurate overwintering estimates, and an inability to link lipid or *Varroa* with individual hives. Furthermore, there was substantial variation between apiaries/beekeepers in overwintering management (insulation, hive placement, mite treatments, overwinter feeding) that could potentially confound our understanding of the causes behind overwintering losses. Thus, the direct relationship of our findings to actual colony overwintering success merits further investigation.

Importantly, there was a significant statistical interaction between landscape cultivation and *Varroa* presence, and the positive effect of low cultivation on lipid content was observed only in colonies free from *Varroa* mite infestation. In colonies infested with mites, there was no difference in lipid stores between bees from different landscapes (Fig 1), so any nutritional benefit due to landscape appears to be lost when mites are present. Furthermore, in bees from low cultivation areas, lipid levels were lower in mite-infested bees compared to mite-free bees, though this difference was not observed in bees from the high cultivation groups (Fig 1). Again, bees seem to receive a benefit in pre-overwintering lipid stores when kept in areas of low landscape cultivation, but only when mites are absent. In bees from high cultivation areas, where lipid content is lower even in the absence of mites, mite infestation did not bring lipid levels even lower. Thus, mite-mediated lipid depletion is not as apparent when lipid levels are already low due to other forms of nutritional stress.

*Varroa* mites represent arguably the most severe stressor on honey bee colonies in the USA and much of the rest of the world, and cause multiple detrimental changes to honey bee physiology, including reduced vitellogenin protein content, hemocyte number, and ecdysteroid hormone titer [20]. Previous work [31] found that there were no differences in lipid levels between healthy newly-emerged honey bee workers and those parasitized by *Varroa* during pupation. Another study found differences in lipid content only in pupal bees from infested versus uninfested colonies, but no differences between newly-emerged adults or foragers [33]. In both of these cases, the authors focused on either individual newly-emerged bees or foragers from a small number of experimental colonies. In the study presented here, the samples were made from a homogenate of approximately 100 workers from the core of a colony during a period in the colony lifecycle where many of the workers should be physiologically winter bees. It is possible that differences in lipid levels are not as evident in newly-emerged or foraging bees, or that differences in lipid levels are more apparent in winter bees, or that differences in lipid content are more observable when comparing a mixture of workers representing the hive as a whole. Conversely, whole-body protein content did not differ between bees from these samples. It is unclear if this is because no protein content differences exist, or if the use of whole-body extracts (which would include large quantities of cuticular proteins), obscured more subtle differences in circulating or fat body protein content ([48, 56]; S1 Fig). Dry body mass also showed no differences, but this has been shown to be a poor indicator of protein content ([57]; S1 Fig).

*Varroa* mite infestation causes other detrimental effects on honey bees, including vectoring of viruses [30]. As shown previously [43, 58, 59], colonies infested with *Varroa* had significantly higher levels of DWV (Fig 2C). This pattern was observed independent of landscape, showing that, even though a lower cultivation landscape may provide nutritional benefits, the presence of mites may still increase virus titers. Landscape cultivation level also had no significant effect on the levels of BQCV, DWV, IAPV, or SBV (Fig 2A, 2B and 2D–2H). However, the lack of differences may relate to the fact that overall virus levels from apiaries in our study were somewhat low. Due to variation in titration methodology and virus dynamics, it is often difficult to put virus loads into biological context. In Locke et al. [60], where similar virus titration methods to ours were used, some hives were allowed to become highly infested with *Varroa* mites, and then died in the winter. Compared to these dying colonies, hives sampled here had substantially (multiple orders of magnitude) lower virus titers, so it is unlikely that the virus titers observed here directly caused colony mortality.

Overall, this study provides new information on how the landscape surrounding bee hives can affect the nutritional physiology of the hives, and also shows that there may be some interaction between the level of landscape cultivation and the presence of *Varroa* mites, at least on lipid content. There has been substantial recent interest in landscape use and nutrition availability as an important factor in honey bee health [4, 16], and recent studies have shed light onto how different landscape types can affect honey bee pollination services [61], foraging behavior [35], pollinator densities [62], colony buildup and food accumulation [38], honey production [37], and beebread nutrition [34]. Our results are novel because they suggest the landscape around a hive also has the potential to affect worker nutritional physiology, particularly in a highly-managed agricultural landscape.

Previous studies have shown contradictory conclusions about the relationship between landscape cultivation and bee health–in some regions, lower landscape cultivation results in ‘better’ metrics of bee health and production [34, 37], while in other areas, more agricultural land is associated with stronger hive development [38]. While the study presented here shows further evidence that bees benefit from less cultivation, it is still worth noting that variability in land use is much more complex than simply cultivated versus non-cultivated land, especially when extrapolating past the local scale. In addition to variability due to landscape use, it is also clear that *Varroa* infestation needs to be taken into account, which has not always been done (e.g., [37, 38]). Here, the presence of *Varroa* significantly interacted with landscape type, obscuring the effects of landscape on nutritional physiology and increasing virus titers, suggesting that some of the benefits of improving the foraging landscape would be lost without *Varroa* control. Overall, while this study leaves many open questions, it also opens doors to future studies that can help elucidate both how landscape affects honey bee physiology and how landscape-mediated nutritional stress interacts with parasite and pathogen infection to affect colony health.

## Supporting Information

S1 Fig Relationship between landscape cultivation and protein levels and dry mass, in presence or absence of *Varroa* mite. Mean +/- SE of whole body protein concentration (A, B) and average dry mass (C, D) of bees from hives in low and high cultivation areas (A, C) and with *Varroa* mites absent or present (B, D). Number of hive sampled indicated, N.S. notes no significant differences between groups.
(TIF)

S1 Table Full dataset used for all analysis, containing anonymized beekeeper code, hive code, landscape composition for all apiaries, and all data associated with each hive. (XLSX)
