# Supplementary figures and images for: Intensively Cultivated Landscape and Varroa Mite Infestation Are Associated with Reduced Honey Bee Nutritional State
Source: PLoS One. 2016 Apr 12;11(4):e0153531. doi: 10.1371/journal.pone.0153531 (PMC4829173; doi:10.1371/journal.pone.0153531)

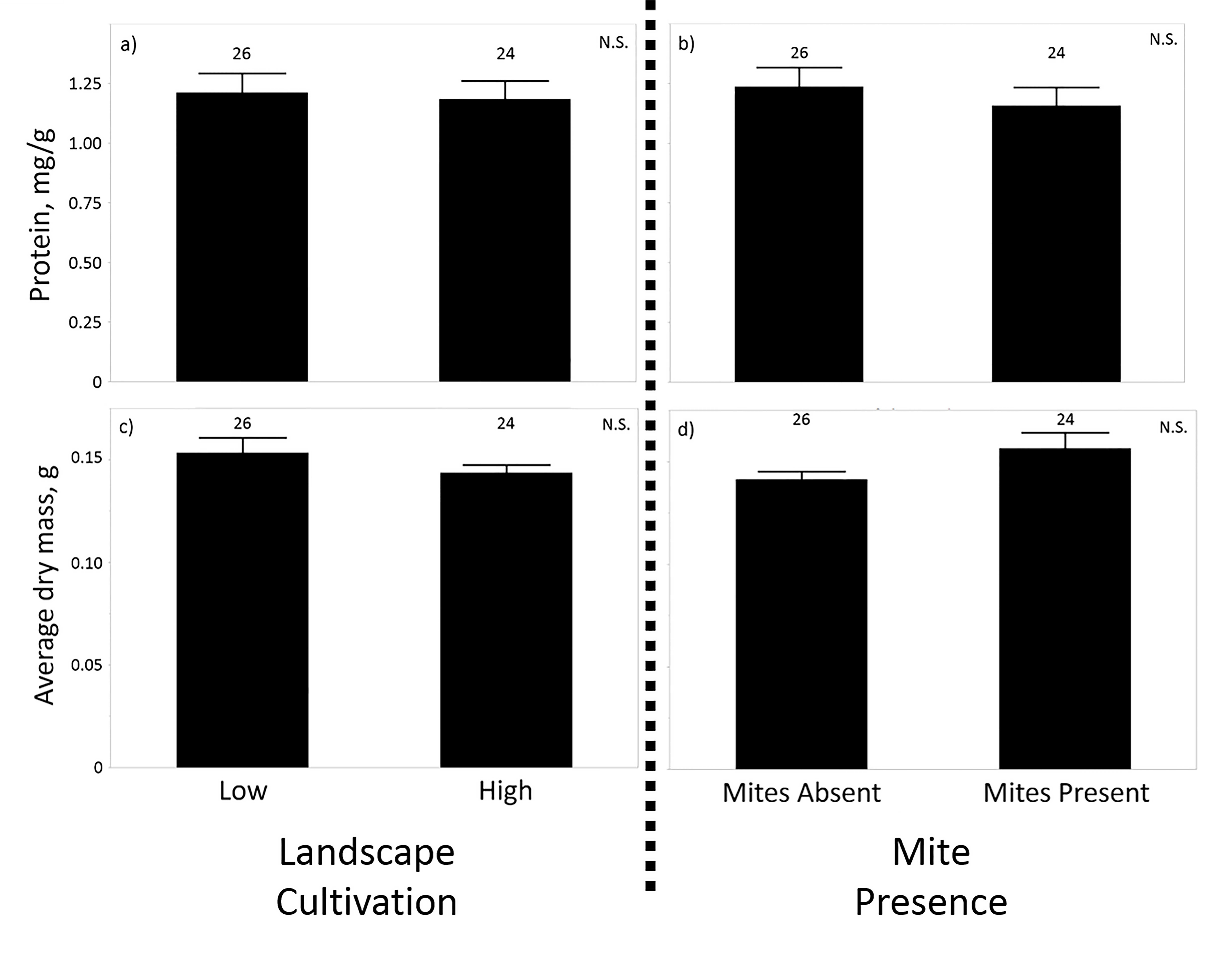

Supplement: S1 Fig — Mean +/- SE of whole body protein concentration (A, B) and average dry mass (C, D) of bees from hives in low and high cultivation areas (A, C) and with Varroa mites absent or present (B, D). Number of hive sampled indicated, N.S. notes no significant differences between groups. (TIF) [file pone.0153531.s001.tif]
